# Everolimus treatment in advanced solid tumors: a personal view

**DOI:** 10.4155/fso.14.1

**Published:** 2015-11-01

**Authors:** Sara Pusceddu, Alice Indini, Giuseppe Procopio

**Affiliations:** 1 Department of Medical Oncology, Unit 1 & ENETs Center of Excellence, Fondazione IRCCS Istituto Nazionale Tumori, Milan, Italy; 2 SS Oncologia medica Genitourinaria, Fondazione Istituto Nazionale tumori Milan

**Keywords:** breast cancer, everolimus, neuroendocrine tumour, renal cell carcinoma

Everolimus is currently approved in four settings: progressive well differentiated pancreatic neuroendocrine tumors (pNETs), metastatic renal cell carcinoma (mRCC), in patients who failed a treatment with tyrosine kinase inhibitor; advanced hormone receptor positive HER-2 negative breast carcinoma in combination with exemestane; and astrocytoma associated with tuberous sclerosis [1]. The approval of everolimus was based on the results of three Phase III, randomized, placebo-controlled trials: RADIANT-3, RECORD 1 and BOLERO 2 [2–4], which included patients with pNET, mRCC and breast carcinoma, respectively. Overall, everolimus was shown to increase the median progression-free survival, the primary endpoint of each study. The safety profile of everolimus was satisfactory: most adverse events were of mild-to-moderate severity and were successfully managed with medical treatment.

Given the activity of everolimus demonstrated in progressing patients affected from pNETs, mRCC and breast cancer, we believe that further prospective clinical trials in earlier settings should be planned. Despite some conflicting findings [5,6], the mechanism of action and the favorable safety profile of everolimus might suggest the possibility of an earlier administration. However, caution should be exerted as an earlier treatment may result in a longer exposure to the molecule potentially leading to the development of cumulative toxicities.

The intermittent use of everolimus, for instance in long-responding patients with mRCC or breast cancer, is also worth investigation in our opinion, since clinical course of the above-mentioned tumors is frequently indolent or only slow-progressing. An intermittent approach would likely be associated with a more favorable safety profile and a reduced burden to the healthcare system. Similarly, the efficacy and safety of everolimus at reduced doses may be evaluated in clinical trials, as it may useful in particular for patients who maintained disease control for a long time.

The optimal ‘stopping-rule’ for everolimus therapy is another clinical issue frequently encountered in daily practice. Does everolimus treatment need to be interrupted as soon as when disease progression is documented? Or should the benefits in terms of pain and quality of life (QoL) associated with everolimus be taken into primary consideration, independently from clinical response? As clinicians with some experience in the use of everolimus, we suggest that a comprehensive evaluation of all benefits associated with a given treatment – including those reported in QoL – is crucial in guiding decision on whether or not to interrupt a therapy. Although at present no data support the use of everolimus beyond progression, we think that – of course in absence of any suitable alternative and as long as improved QoL is observed – patients should not be denied the possibility to continue everolimus treatment also beyond progression of disease.

A possible limitation to a more widespread use of everolimus in clinical practice – including its potential administration using alternative dosing strategies – lies in the overall paucity of data collected in the ‘real world’ population [7]. Some observational controlled studies have been conducted to characterize the effectiveness and clarify the safety profile of everolimus in clinical practice [8–10], but further evidence in this setting appears required [7]. In particular, information on any potential predictive factor appears warranted. The Phase IV ORCHIDEE study, currently in progress, is specifically designed to identify factors predictive of outcome in mRCC patients on second-line everolimus [7]; this study represents one of the multiple efforts aimed at further characterize the use of everolimus in clinical practice and gain additional experience on the use of this effective treatment.
